# Proximal partners of the organellar N-terminal acetyltransferase NAA60: insights into Golgi structure and transmembrane protein topology

**DOI:** 10.1098/rsob.240225

**Published:** 2025-02-19

**Authors:** Sebastian Tanco, Veronique Jonckheere, Arun Kumar Tharkeshwar, Annelies Bogaert, Kris Gevaert, Wim Annaert, Petra Van Damme

**Affiliations:** ^1^ iRIP Unit, Laboratory of Microbiology, Department of Biochemistry and Microbiology, Ghent University, Ghent, Belgium; ^2^ VIB Center for Medical Biotechnology, VIB, Ghent, Belgium; ^3^ Departament de Bioquímica i Biologia Molecular, Universitat Autònoma de Barcelona, Cerdanyola del Vallès, Barcelona, 08193, Spain; ^4^ Department of Neurosciences, KU Leuven, Leuven, Belgium; ^5^ Laboratory for Membrane Trafficking, VIB-Center for Brain and Disease Research, Leuven, Belgium; ^6^ Department of Biomolecular Medicine, Ghent University, Ghent, Belgium

**Keywords:** proximity-dependent biotin identification (BioID), Golgi fragmentation, NAA60, N-terminal acetylation, proxeome, transmembrane protein topology

## Abstract

Biotin identification (BioID) is an interactomics approach that utilizes proximity labelling to map the local interactome or proxeome of proteins within a cell. This study applies BioID to investigate proteins proximal to NAA60 (N-alpha-acetyltransferase 60), an N-terminal acetyltransferase (NAT) of pathological significance in human disease, characterized by its unique Golgi localization. NAA60 is known to N-terminally acetylate transmembrane proteins that present their N-terminus on the cytosolic face of the membrane, and its involvement in maintaining Golgi structure has previously been established. Using a stable cell-line expressing an NAA60-BirA* fusion protein, we isolated biotinylated proteins through streptavidin affinity purification. Mass spectrometry analysis revealed over 100 proximal partners of NAA60, enriched in proteins localized on the *trans*-side of the Golgi apparatus. High-confidence proximity interactors included golgins and GRASP proteins, essential for Golgi integrity. Considering the transmembrane nature of NAA60, the identification of biotinylated peptides inferred the topology of transmembrane protein interactors within the secretory pathway. Subsequent suborganellar localization analysis revealed a more prominent *medial*/*trans*-Golgi localization of NAA60. Our findings underscore the role of NAA60 and its interactors in maintaining Golgi structural integrity and highlight the effectiveness of BioID in generating critical protein topology data, invaluable for enhancing the prediction of protein topology within cellular compartments.

**Figure:**
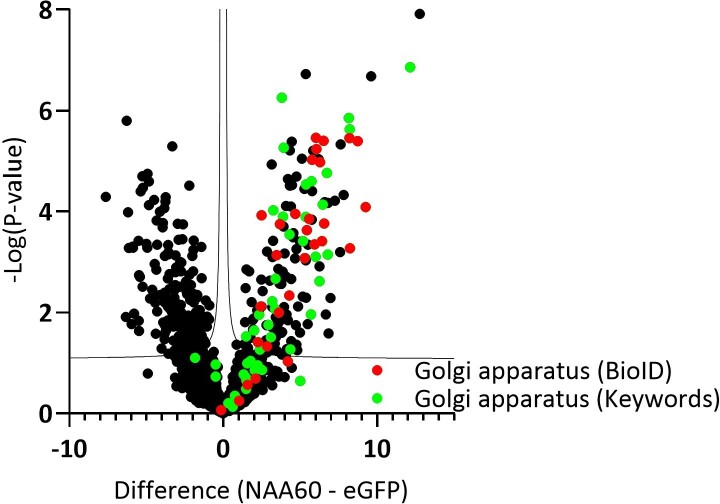

## 1. Background

Eukaryotic protein biogenesis relies on multiple factors that dynamically interact with the translating ribosome, ensuring nascent polypeptides are correctly processed, translocated into various subcellular compartments and folded into their native structure [1]. These factors include modifying enzymes, chaperones and targeting complexes. In this context, the ribosome serves as a central hub, coordinating all these co-translational processes both temporally and spatially. Among the enzymatic modifications occurring during translation are N-terminal acetylation (Nt-acetylation), cleavage of the initiator methionine (iMet) by methionine aminopeptidases (MetAPs) and signal sequence removal.

Nt-acetylation stands out as one of the most prevalent protein modifications in eukaryotes; proteomic analyses have revealed that approximately 50–90% of proteins in yeast, plants, fruit flies and humans undergo Nt-acetylation [2,3]. Primarily assumed to occur co-translationally, Nt-acetylation is catalysed by ribosome-associated N-terminal acetyltransferases (NATs), which transfer an acetyl moiety from acetyl coenzyme A (Ac-CoA) to the primary α-amino group of a nascent polypeptide. The narrow dimension of the ribosome exit tunnel limits large domain folding of the nascent chain, thereby creating a window of opportunity for protein modifications as the nascent chain emerges from the ribosome.

Despite its prevalence, the functional implications of Nt-acetylation remain incompletely understood. Initially, Nt-acetylation was thought to protect proteins from degradation [4]. While more recent evidence suggests that Nt-acetylation may confer protective effects and is associated with age-dependent longevity and motility [5], other studies indicate that it can promote the degradation of certain proteins through the Ac/N-degron pathway, which mediates the recruitment of specific ubiquitin ligases [6,7]. Furthermore, Nt-acetylation has been shown to influence the formation of protein complexes, as exemplified by its effect on NEDD8 ligation enzymes [8], and has been linked to prion formation [9]. Nt-acetylation has also been implicated in the specific targeting of proteins to membranes of the nucleus [10], Golgi [11], vesicles [12] and lysosomes [13]. However, taken together, the evidence does not support a general role for Nt-acetylation in protein targeting or degradation.

To date, eight NATs (NAA10-NAA80) have been identified in higher eukaryotes. These NAT enzymes differ in subunit composition (if part of a complex) and substrate specificity [14,15]. The N-terminal amino acid sequence of a protein, specifically the initial N-terminal amino acids, is the primary determinant of whether a protein is Nt-acetylated and by which NAT [16], although some redundancy among the NATs has been observed [3,17].

In this study, we focus on NAA60, one of the most recently identified members of the NAT family. Previously, we reported NAA60 (also known as NatF) as a NAT specific to higher eukaryotes, contributing to the higher level of Nt-acetylation observed in human proteomes compared with those of yeast [3]. NAA60 targets initiator methionine (iMet)-hydrophobic or iMet-amphipathic N-termini [3].

We discovered that NAA60 differs from most previously characterized NATs due to its specific organellar localization, associating with the Golgi apparatus, where it resides on the cytoplasmic face of the membrane, thereby representing a peripheral membrane protein [18]. Similarly, NAA70, a plastid-specific NAT, has been characterized in *Arabidopsis thaliana* and found to reside in chloroplasts [15]. Our research indicates that NAA60 primarily acts on the N-termini of transmembrane protein substrates that face the cytosolic side of intracellular membranes and are thus known or predicted to have an N-in (inside) topology [19]. This aligns with the findings on NAA60’s cytosolic accessibility, establishing Nt-acetylation as a common modification among transmembrane proteins—a thus far poorly characterized component of the Nt-acetylome [19].

Recent insights into the role of NAA60 have illuminated its significance in primary familial brain calcification (PFBC) and its potential implications for human disease pathways [20]. Biallelic loss-of-function mutations in *NAA60*, directly linked to autosomal recessive PFBC, highlight its critical role in the proper functioning of the nervous system. These mutations impair NAA60’s Nt-acetylation capacity and/or stability and result in the mislocalization and potential malfunctioning of various membrane proteins. Notably, this includes reduced surface levels of the phosphate importer SLC20A2, resulting in disrupted phosphate and calcium homeostasis within the brain [20], an observation in line with proteomics findings showing altered abundances of various transmembrane proteins in the organellar-enriched fraction of si*NAA60* knockdown cells [19]. These discoveries unveil a broader involvement of NAA60 in cellular transport processes and membrane stability, particularly at the Golgi apparatus and position NAA60 as a potential therapeutic target in neurodegenerative diseases.

In vertebrate cells, the Golgi apparatus consists of stacked cisternae interconnected by tubules and vesicles, forming a compact structure known as the Golgi ribbon. The Golgi ribbon is widely recognized as a requirement for cell polarization, particularly in specialized cells that depend on polarized secretion for advanced functions. Examples include the directional cell migration of fibroblasts [21], the regulation of dendritic growth in neurons [22] and the polarized delivery of secretory granule granules by cytotoxic T cells [23]. Disruption of human NAA60 induces Golgi fragmentation, resulting in smaller, dispersed substructures, underscoring NAA60’s vital role in maintaining Golgi integrity [19,20].

In this study, we exploited BioID (proximity-dependent biotin identification) proximity labelling to comprehensively investigate the proximal partners of NAA60, for the first time shedding light on its suborganellar whereabouts. Through this approach, we identified potential interactors and regulators of NAA60’s function, which may contribute to the Golgi-fragmentation observed in NAA60-associated conditions. Furthermore, our electron microscopy studies in *NAA60* knockout (KO) cells provided detailed ultrastructural evidence of Golgi fragmentation, further reinforcing the importance of NAA60 in maintaining Golgi integrity. These findings collectively suggest potential disruptions in NAA60 complex formation and altered localization of its proximal interactors, emphasizing the broader functional significance of NAA60 beyond Nt-acetylation. Overall, our study contributes to a deeper understanding of NAA60 biology and its implications in cellular processes and disease pathways.

## 2. Results

### 2.1. BioID of N-alpha-acetyltransferase 60 reveals Golgi proximal proteins

To identify the proximal neighbours (or ‘proxeome’) of human NAA60, we made use of BioID, a proximity-dependent biotin identification strategy that allows the labelling of proteins located near BioID-fused NAA60, capturing (transient) protein–protein interactions while preserving spatial information about these interactions. BioID uses a mutated form of the *E. coli* biotinylase (R118G, hereafter referred to as BirA*) that is capable of promiscuous proximity-dependent biotinylation [24]. Biotinylated proteins proximal to NAA60 were isolated using streptavidin affinity capture and identified through tandem mass spectrometry (MS/MS) [24].

Upon transfection of BirA*-tagged NAA60, co-visualization analysis using fluorescence microscopy showed that the biotinylated protein pattern associated with NAA60-BirA* protein expression displays an organellar distribution consistent with the previously reported Golgi localization of NAA60 [19] and demonstrates partial co-localization with the *cis*-Golgi marker GM130 (electronic supplementary material, figure S1A).

**Figure 1.**
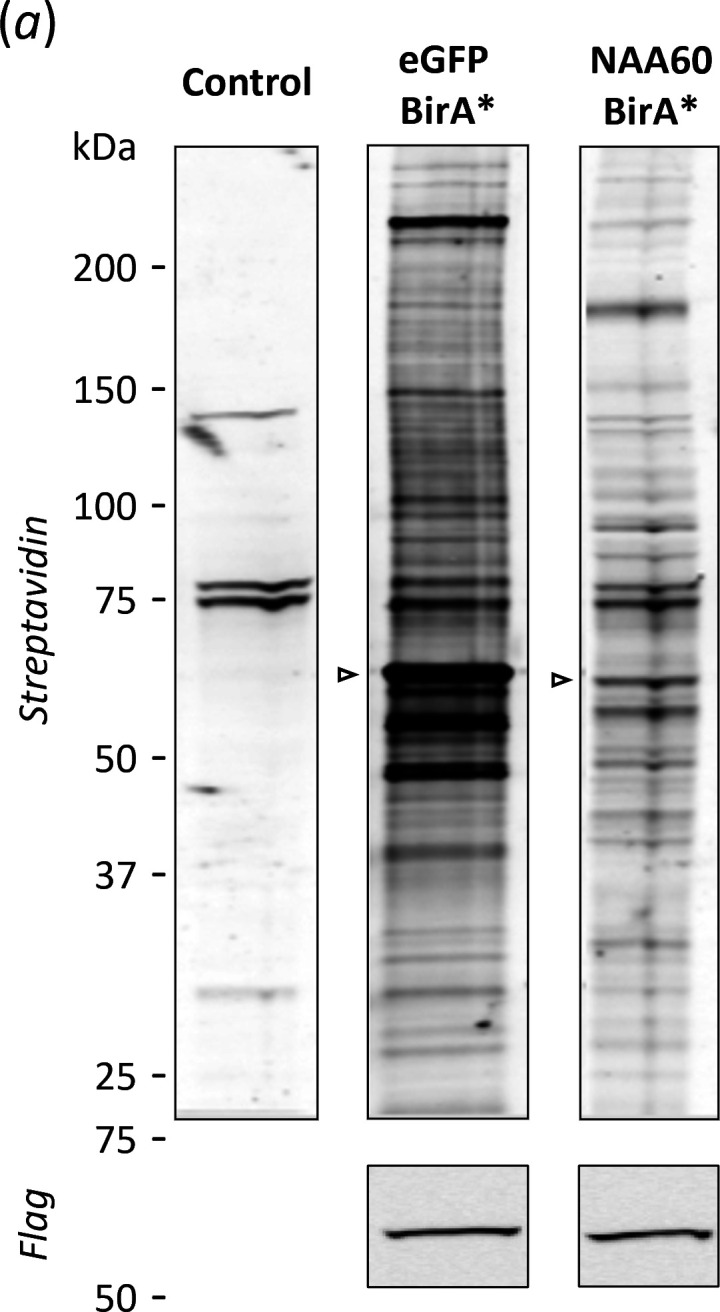
Comparison of NAA60-BirA* and BirA*-eGFP protein biotinylation. Immunoblot analysis of biotinylated protein and NAA60 expression of stable human Flp-In™ T-REx™-293 cell lines expressing NAA60-BirA* or BirA*-eGFP. Total lysates were probed with streptavidin-Alexa Fluor™ 680 conjugate and anti-Flag antibody. Open arrows indicate *cis*-biotinylation of eGFP- and NAA60-BirA* fusion proteins.

Evaluation of protein extracts from corresponding stable (doxycycline-induced) human Flp-In™ T-REx™−293 cell lines expressing NAA60-BirA* or BirA*-enhanced green fluorescent protein (eGFP)—serving as control, supplemented with biotin for 24 h, revealed that NAA60-BirA* exhibits a distinct pattern of protein biotinylation compared with the control BirA* fusion protein eGFP-BirA* (figure 1).

Following labelling, biotinylated proteins were isolated using streptavidin purification, the proteins were digested and the resulting peptides were subjected to liquid chromatography with tandem mass spectrometry (LC-MS/MS). To enable relative comparisons of the proxeomes, label-free quantification was performed using the intensity-based absolute quantification (iBAQ) values obtained with the iBAQ algorithm [25]. After log2 transformation of the iBAQ values, both Pearson correlation (electronic supplementary material, figure S2A) and principal component analysis (PCA) (electronic supplementary material, figure S2B) demonstrated high correlation and clustering among replicate samples, while highlighting the variability between the different BioID set-ups analysed. A two-sample *t*‐test analysis was performed to reveal (differential) NAA60 interactors. Out of the 758 human protein entries with at least three valid values in one of the set-ups analysed, based on a permutation-based false discovery rate (FDR) calculation (FDR of 0.05 and an S0 of 0.1), 111 proteins with increased abundance in the NAA60 compared with the eGFP BioID control set-up were identified (electronic supplementary material, table S1). It is notable that among the few previously reported binary NAA60 interactors (six reported in IntAct; https://www.ebi.ac.uk/intact/home), including the peripheral membrane protein Endophilin-B1 identified by validated yeast-two-hybrid [26], we identified its homologue Endophilin-A2 (44% homology, 27% identity), previously found to be Golgi associated [27], as a putative NAA60 interactor (electronic supplementary material, table S1), representing a first indication of the specificity of the NAA60 proximal partners identified.

Intriguingly, the pool of NAA60-proximal proteins, representing putative NAA60 interactors, encompasses a variety of regulatory proteins governing crucial aspects of vesicular trafficking and secretion, in addition to Golgi integrity maintenance. Specifically, this includes numerous golgins (GOLGA2 or GM130, GOLGA3, GOLGA4, GOLGA5, GOLGB1 and GORAB), the pivotal structural Golgi protein GORASP2 or GRASP55 (Golgi apparatus Golgi reassembly stacking protein 2), as well as membrane-tethering proteins, such as thyroid receptor-interacting protein 11 (TRIP11) and synaptosomal-associated protein 23 (SNAP23). Moreover, several small GTPases from the Rab family (RAB1A, RAB7A, RAB18, RAB21 and RAB6A), including the transport regulator from early to late endosomes, Rab7A [28,29], were identified as NAA60 proximal proteins. Additionally, GTPase-activation proteins (GAPs) regulating Arf (ADP-ribosylation factor)-family small GTPases, such as ARFGAP2 (ADP-ribosylation factor GTPase-activating proteins 2) and ARFGAP3, key regulators of intracellular trafficking and Golgi structure, were identified. Furthermore, SNARE proteins, which are crucial for vesicle fusion, including five vesicle-associated membrane proteins (VAMPs) (VAMP2, VAMP3, VAMP8, VAPA and VAPB), SNAP23 and 29 and Golgi SNAP receptor complex member 1 (GOSR1), were found to be among the NAA60 proximal partners (electronic supplementary material, table S1).

**Figure 2.**
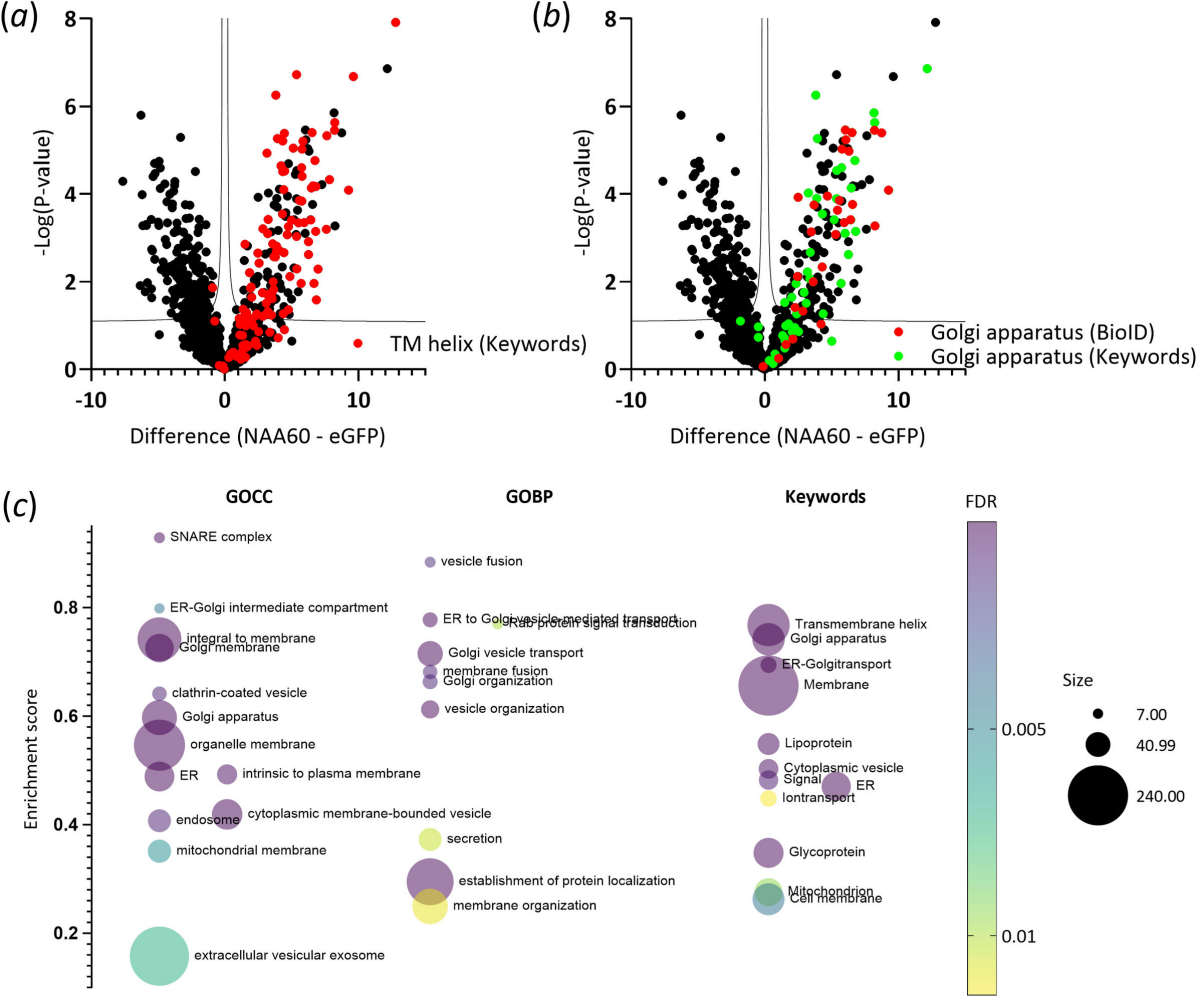
Analysis of the NAA60 proxeome using BioID. Volcano plots highlighting transmembrane (*a*) and Golgi proteins (*b*) corresponding to NAA60-enriched annotations. Volcano plots depict the *t*‐test results for 758 human protein entries, each with at least three valid values in one of the set-ups analysed. These plots highlight significant data points based on a permutation-based FDR calculation, illustrating proteins with increased abundance in the NAA60 compared with the eGFP BioID control set-up. Two-sample *t*-tests were performed with an FDR of 0.05 and an S0 of 0.1, represented by a solid black line. The plots display log2-transformed iBAQ intensities on the *x*-axis and -log10 (*p*‐value) on the *y*-axis. Specific focus is on proteins associated with 'TM Helix' (*a*) and 'Golgi apparatus' (*b*), either as keywords or as retrieved annotations from the BioID-based proximity map of human HEK293 cells (available at https://cell-map.org/ and [31]) (electronic supplementary material, table S1). (*c*) Multiple-variable bubble plot of significantly regulated proteins (FDR < 1%) showing normalized positive enrichment scores (0.1 to +0.95 max) for selected keywords and Gene Ontology (GO) categories (including Cellular Component (GOCC) and Biological Process (GOBP)), comparing NAA60 to eGFP (electronic supplementary material, table S3). Term enrichment was determined using the 1D annotation enrichment algorithm embedded in the Perseus software suite [30], with *p*-values corrected for multiple hypothesis testing using the Benjamini–Hochberg FDR. Only terms with corrected *p*-values < 0.02 were included. Bubble size indicates the number of regulated proteins associated with a given keyword or GO term, and the colour gradient represents the *p*-value scale.

For a broader perspective on the relative enrichment of gene annotations within the category of NAA60 proximal partners, we included UniProt keywords, Gene Ontology Cellular Component (GOCC), Gene Ontology Biological Process (GOBP), Gene Ontology Molecular Function (GOMF) names and subcellular localization data. The one-dimensional (1D) annotation enrichment analysis [30] similarly revealed an enrichment of (transmembrane) Golgi proteins implicated in endoplasmic reticulum (ER) to Golgi vesicle-mediated (ion) transport and organization, as well as secretion and other related ontologies (figure 2*c*, electronic supplementary material, table S3). By and large, the NAA60 proximal partners identified by BioID proximity labelling align with the previously reported subcellular localizations and functions attributed to NAA60. Furthermore, they correspond to the transmembrane substrate category associated with NAA60, as previously reported [19].

Remarkably, none of the previously identified 23 NAA60 substrates were identified as proximal NAA60 partners. However, it is noteworthy that the proxeome dataset includes functionally related proteins and homologues of previously identified substrates. Furthermore, comparison between the NAA60 proxeome and previously reported proteomics data revealed that the N-termini of eight NAA60 proximal proteins identified here were significantly regulated in abundance (*p* ≤ 0.05) upon *NAA60* knockdown [19]. Among these, seven were upregulated (Sec22B, Aup1, FAM219a, SLC1A5, Golgb1, Gcp60 and Scps2), while one was downregulated (SLC35E1). This suggests that destabilization or mislocalization of NAA60 proximal partners upon reduced and/or impaired Nt-acetylation capacity of NAA60 following knockdown (electronic supplementary material, table S1).

While our initial screen revealed that the majority of identified proximal proteins are localized to the Golgi and other membranous secretory organelles (electronic supplementary material, table S1, figure 2), the precise suborganellar localization of NAA60 within the Golgi apparatus remains elusive. To address this and potentially differentiate NAA60 proximal partners from physical interactors, we conducted a comparative BioID screen by analysing the proxeomes of two additional Golgi-resident transmembrane proteins: the *medial*/*trans*-Golgi cytidine monophosphate-sialic acid transporter SLC35A1 [32] and the ER-Golgi intermediate compartment and *cis*-Golgi network guanosine diphosphate-fucose transporter membrane protein SLC35C2 [33], alongside NAA60. Immunofluorescence microscopy, performed to evaluate the subcellular localization of the labelled proxeome of the BirA*-fusion proteins, again revealed a distinct streptavidin signal resembling a Golgi-like pattern in all set-ups treated with biotin, suggesting that all fusion proteins localize similarly to their native counterparts. However, while partial co-localization of the biotinylation signal with GM130 can be observed for SLC35A1-BirA*, no GM130 co-localization is observed in the SLC35C2-BirA* setup (electronic supplementary material, figure S1B-C).

**Figure 3.**
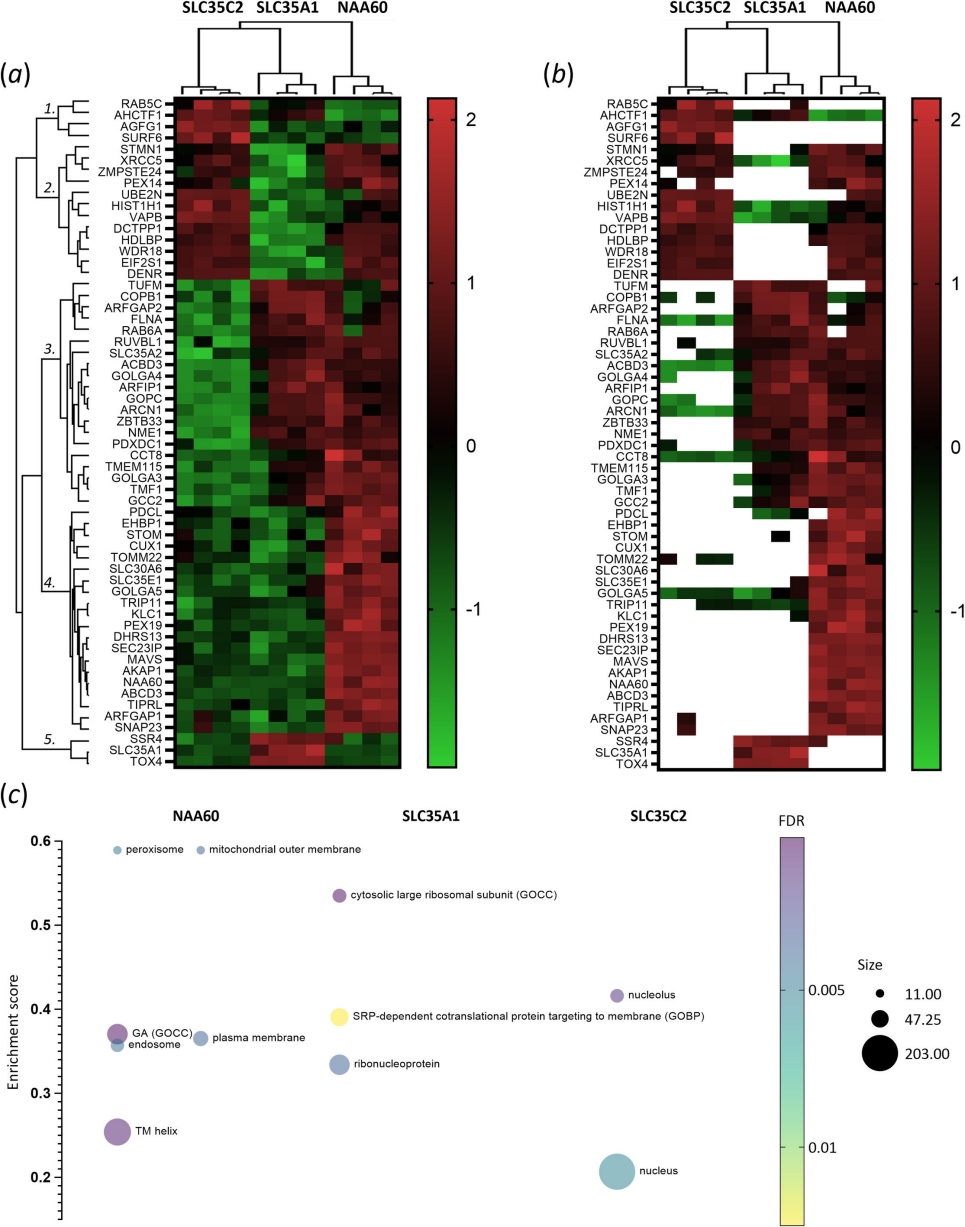
Differential comparison of NAA60, SLC35A1 and SLC35C2 proxeomes. (*a*) Heat map representation of cluster analysis following ANOVA, displaying the intensities of 59 proteins with significantly different abundances (*p* ≤ 0.01) across the interactomes (electronic supplementary material, table S2). Five main clusters emerge, with clusters 1, 4 and 5 predominantly enriched for SLC35C2, NAA60 and SLC35A1 proximal proteins, respectively. (*b*) Similar heat map to (*a*), but with imputed values removed. Colours corresponding to z-scores range from green for low intensities to red for high intensities. (*c*) Multiple-variable bubble plot displaying significant regulated proteins (FDR < 1%) with normalized positive enrichment scores ranging from 0.1 to +0.95 maximum for selected keywords and GO categories (GOCC and GOBP), comparing NAA60 versus SLC35A1 and SLC35C2 set-ups (electronic supplementary material, table S4). Term enrichment was determined using the 1D annotation enrichment algorithm integrated into the Perseus software suite [30], with *p*-values corrected for multiple hypothesis testing using the Benjamini–Hochberg FDR.

In corresponding stable cell lines, optimized doxycycline expression conditions were tailored to each set-up to achieve comparable levels of protein biotinylation (1 μg ml^−1^ for NAA60 and 1.6 and 1.2 ng ml^−1^ doxycycline for SLC35A1 and SLCA35C2, respectively). Biotinylated proteins from biological quadruplicate samples were then isolated and subjected to LC-MS/MS analysis. Upon log2 transformation of the iBAQ values, while clustering was still evident, the Pearson correlation among replicate samples notably decreased compared with the initial BioID set-up comparing NAA60 with its control eGFP (electronic supplementary material, figure S1C). PCA analysis, however, revealed clear clustering among replicate samples (electronic supplementary material, figure S1D). Of the 579 human protein entries with at least three valid values in one of the set-ups analysed, a multiple analysis of variance (ANOVA) test identified 59 (differential) interactors (*p* ≤ 0.01) among the analysed baits (electronic supplementary material, table S2, figure 3*a*–b).

The intensities of proteins with significantly different abundance (*p* ≤ 0.01) in the respective proxeomes (59 proteins in total) are depicted in heat maps (figure 3*a*,*b*). While SLC35C2 was not identified, the NAA60 and SLC35A1 bait sequences exhibited expected enrichment in the respective set-ups. Notably, five main clusters were observed, with clusters 1, 4 and 5 enriched for putative SLC35C2, NAA60 and SLC35A1 interactors, respectively (figure 3*a,b*).

Consistent with the reported bait localizations and initial BioID results, GO-enrichment analysis pointed to an overall enrichment of identified proteins localized to Golgi (membrane), vesicular structures and proteins implicated in ER to Golgi vesicle-mediated transport and regulated exocytosis (GOBP), as well as SNARE proteins (GOCC). In addition, the current set-up enabled the identification of unique proximal partners and distinct GO aspects of the analysed baits (electronic supplementary material, table S4, figure 3*c*).

Cluster 4 comprised 20 proteins exclusively enriched in the NAA60-BirA* set-up (figure 3*a,b*). In addition to expected cytosolic proteins, given the cytosolic localization of the NAA60-BirA* fusion at the Golgi membrane, various mitochondrial, peroxisomal, plasma membrane and endosomal proteins frequently harboring a transmembrane helix were enriched among the exclusive NAA60 proximal proteins. These findings suggest possible additional localizations for NAA60, consistent with previous findings showing co-localization of both endogenous and tagged NAA60 with various markers of the Golgi, as well as components of the secretory and endocytic pathways, including peroxisomes, early endosomes, secretory vesicles and the ER [19]. Together, these findings underscore the multifaceted localization of NAA60 within cellular compartments. Notably, previously identified putative NAA60 proximal partners (compared with the eGFP control), including SNAP23, ABCD3, TRIP11, DHRS13, MAVS, GOLGA5, AKAP1 and SLC35E1, were also exclusively enriched in the NAA60-BirA* set-up, likely signifying unique NAA60 proximal partners or direct physical NAA60 interactors (electronic supplementary material, tables S1 and S2). Of note, TRIP11 and Golgin subfamily A member 5 are both implicated in Golgi organization and Golgi stack and ribbon formation, potentially linking these NAA60 interactors with the observed Golgi-fragmentation phenotype [19,20].

‘Ribonucleoprotein’, ‘Ribosomal protein’ and ‘SRP-dependent cotranslational protein targeting to membrane’ were enriched annotation terms in the SLC35A1 set-up, while abundances of identified nuclear and nucleolar proteins were generally higher in the proxeome samples of SLC35C2 (electronic supplementary material, table S4, figure 3*c*). Notably, in line with the co-localization observed with GM130 (electronic supplementary material, figure S1), there was an increased correlation and tighter clustering between the (enriched) proxeomes of NAA60 and SLC35A1 compared with SLC35C2, as indicated by the average iBAQ correlations and non-supervised hierarchical clustering analysis, visualized in figure 3 and S2C-D. This, along with the annotation enrichment and pairwise analysis conducted (data not shown), suggests that NAA60 predominantly localizes to the *medial*/*trans*-Golgi compartment, while *cis*-Golgi and ER markers are more enriched in the case of SLC35C2. Additionally, the pairwise comparison of NAA60 with SLC35A1 interactors suggests that the SLC35A1 proxeome is enriched in ER proteins, implying that NAA60 spends comparatively less time at the ER. While these findings do not preclude a possible localization of NAA60 at the ER, they support a more prominent localization of NAA60 in the Golgi (and later parts of the secretory pathway). All enriched GO terms (1D annotation enrichment analysis, corrected *p*-values of <0.01) when comparing NAA60 versus SLC35A1 and SLC35C2 set-ups are depicted in figure 3*c* and listed in electronic supplementary material, table S4.

**Figure 4.**
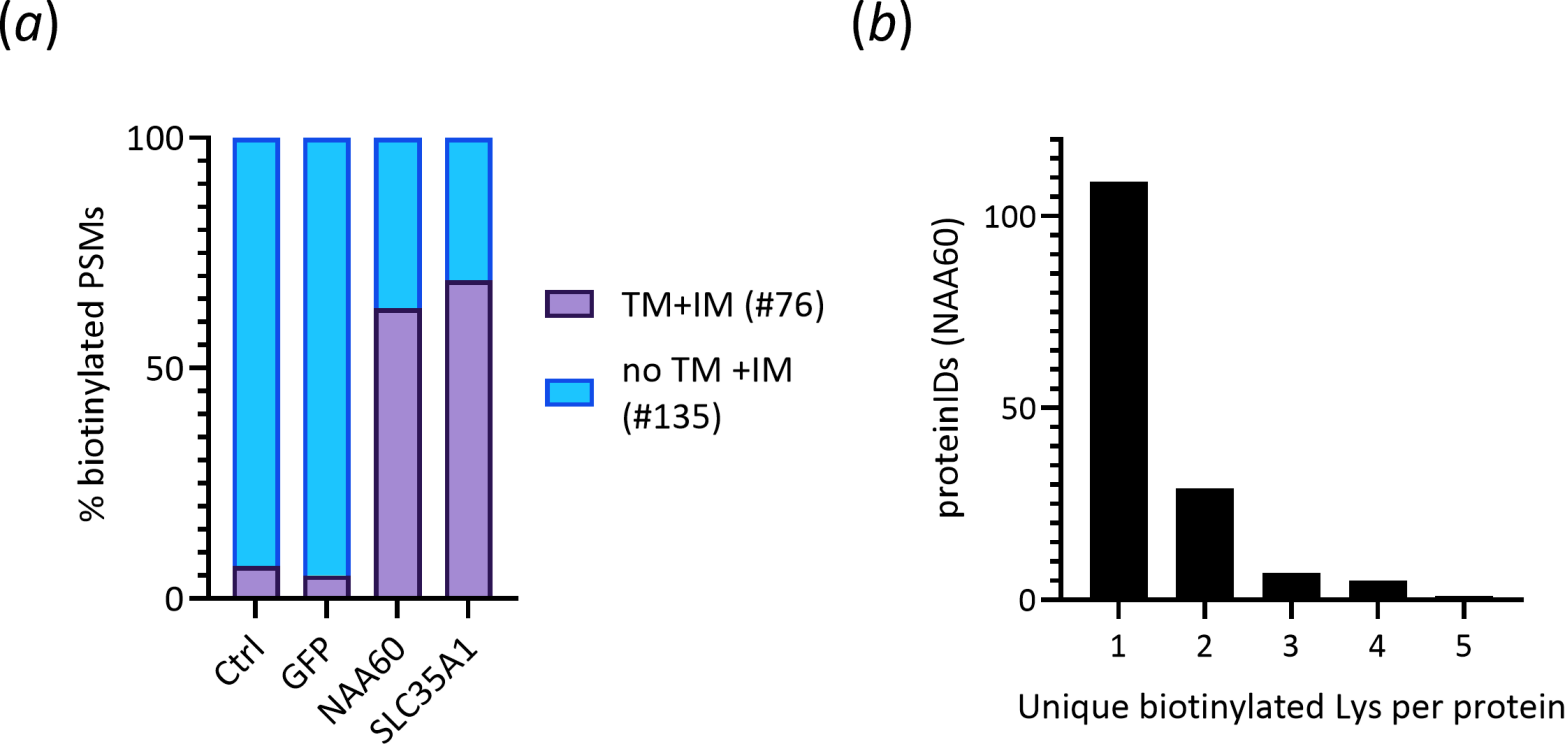
Identification of biotinylated peptides after post-on-bead trypsin digestion peptide elution. (*a*) The percentage of peptide-spectrum matches (PSMs) assigned to transmembrane (TM) or integral membrane (IM) proteins following the identification of biotinylated peptides in the control, NAA60 and SLC35A1 set-ups (highlighted in purple). (*b*) Protein identifications and their corresponding unique biotinylation sites identified in the NAA60 samples analysed.

### 2.2. BioID as a valuable source of topological information

Conventionally, biotinylated proteins captured using BioID are subjected to on-bead trypsin digestion followed by MS analysis. However, the grand majority of the biotinylated peptides remain bound to the streptavidin resin and are, consequently, not directly analysed. Yet, these peptides serve as valuable proxies for protein interactions, aiding to identify likely more direct interactors. Additionally, they may contain crucial information about the interaction sites, providing further insight into the nature of the protein interactions. Moreover, for transmembrane proteins biotinylated by NAA60-BirA*, the cytosolic location of the BirA* fusion at the C-terminus of NAA60 [18] implies that biotinylated peptides from integral membrane proteins should point directly to the cytosolic loops and cytosolic protein termini of proximal NAA60 proteins. To enrich and detect biotinylated peptides, we used a biotinylated peptide elution strategy post on-bead trypsin digestion [34]. Specifically, biotinylated peptides were eluted from the control eGFP and NAA60 replicate samples from the comparative eGFP versus NAA60-BirA* BioID screen, as well as from the NAA60 and SLC35A replicate samples from the second BioID screen, with control HEK293 cells serving as an additional control. Following LC-MS/MS analysis, a total of 849 peptide-spectrum matches (PSMs) corresponding to biotinylated peptides were identified, with 513 identified in the NAA60 samples and 125, 170 and 41 in the SLC35A1, eGFP and HEK293 control set-ups, respectively (electronic supplementary material, table S5). As anticipated, the identification of biotinylated peptides originating from naturally biotinylated carboxylases, such as pyruvate carboxylase (PC), propionyl-CoA carboxylase alpha chain (PCCA) and mitochondrial methylcrotonoyl-CoA carboxylase subunit alpha (MCCC1), constituted a significant portion of the PSMs identified in the control set-ups, with peptides from PC and PCCA alone accounting for more than half of the biotinylated PSMs identified (electronic supplementary material, table S5). Additionally, four unique *cis*-biotinylation sites were identified in the BirA* sequence (electronic supplementary material, table S5). Protein identification analysis revealed an over 10-fold enrichment of transmembrane proteins in the NAA60 and SLC35A1 samples compared with the controls (i.e. 62% and 69% versus 5–7% of proteins) (figure 4), corroborating our BioID results.

**Figure 5.**
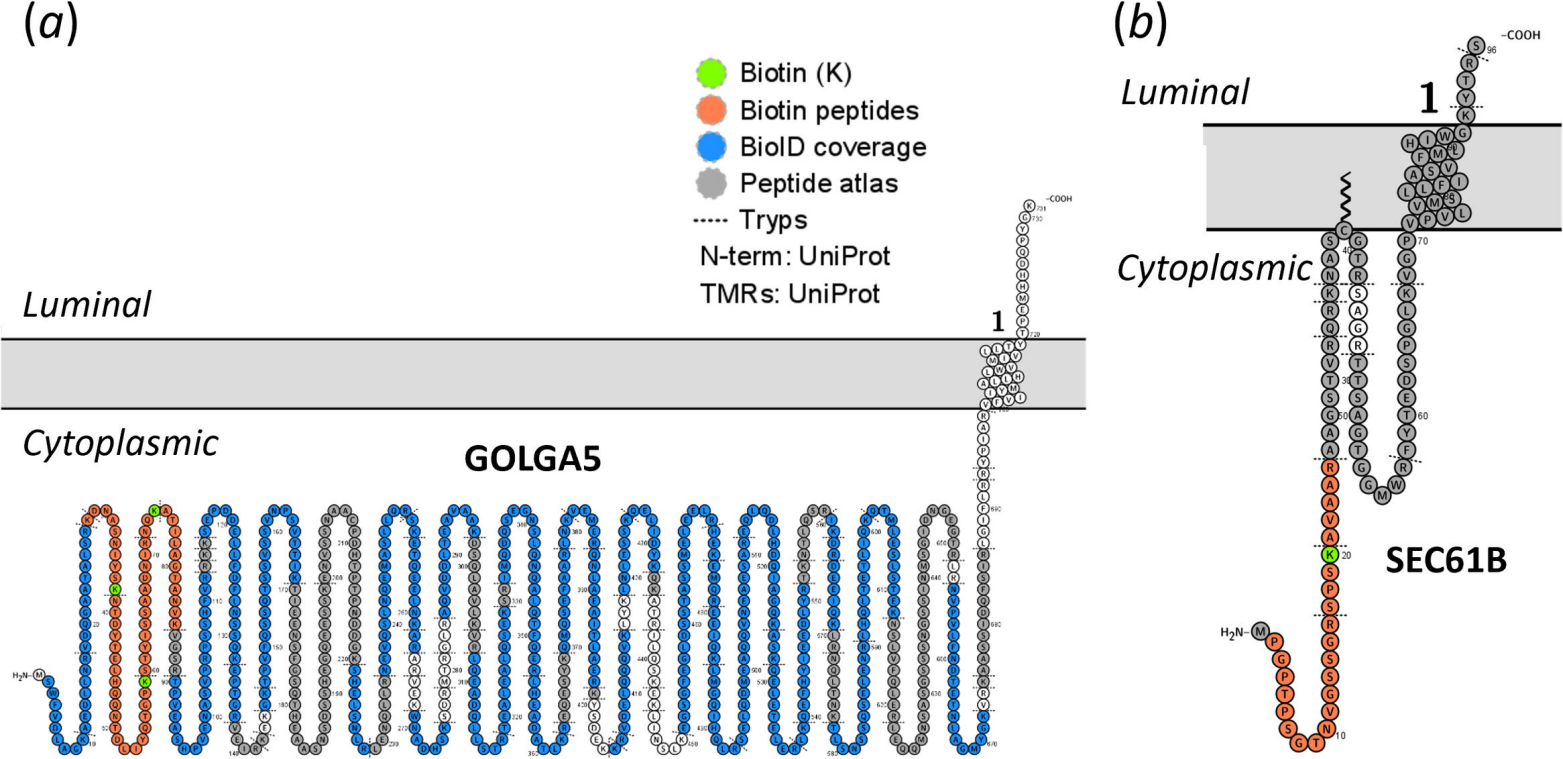
Membrane topology visualization of experimentally identified peptides from NAA60 proximal partners, golgin subfamily a member 5 and protein transport protein Sec61 subunit beta. Protein sequences and transmembrane topology were automatically retrieved from UniProt and visualized using Protter [35]. Theoretical tryptic cleavage sites are indicated by dashed lines positioned vertically onto the primary protein sequence. BioID-identified peptides and biotinylated peptides are highlighted in blue and orange, respectively, with Lys-biotinylation sites indicated in green ((*a*) GOLGA5: K38, K59 and K74 and (*b*) SEC61B: K20). The identified biotinylated peptides are highly likely to originate from cytoplasmic-exposed protein domains, and their position relative to the membrane can provide experimental data of transmembrane topology and protein interaction. Similarly, for the protein transport protein Sec61 subunit beta (SEC61B), a component of the SEC61 channel-forming translocon complex involved in ER membrane insertion of transmembrane proteins and previously shown to be regulated in expression upon si*NAA60* knockdown [19], a unique biotinylation site (*k20*) was identified in both the NAA60 and SLC35A1 set-ups (electronic supplementary material, table S5, (figure 5*b**b*). While the structure of SEC61B has been resolved with an N-in/C-out topology, topology prediction models present conflicting results, with both N-in/C-out (Philius, SCAMPI) and N-out/C-in (TOPCONS, (SP)OCTOPUS) configurations predicted. This underscores the value of using biotinylation data to refine and improve topology predictions and protein annotations.

The 513 biotinylated PSMs identified in the NAA60 samples corresponded to 151 unique biotinylated peptides, covering 213 unique biotinylation sites. Among these, 109, 29, 7, 5 and 1 protein identifications harboured 1–5 biotinylation sites, respectively (figure 4*b* and electronic supplementary material, table S5). Significantly, of the 111 proteins with increased abundance in the NAA60 set-up compared with the eGFP BioID control, biotinylated peptide evidence was found for approximately half of them (52/111), further substantiating their proximity and possible (indirect) interaction with NAA60 (electronic supplementary material, tables S1 and S5). As an illustrative example, five PSMs matching three unique biotinylated peptides from GOLGA5 (K38, K59 and K74) were exclusively identified in the NAA60 set-up, consistent with previous findings of exclusive enrichment in the NAA60 BioID samples. GOLGA5 is an integral membrane protein with a single transmembrane domain close to its C-terminus, displaying an N-in (cytoplasmic) and C-out (luminal) orientation. Aligned with the cytoplasmic localization of BirA* fused to NAA60, the three biotinylation sites identified reside in the disordered region preceding the coiled-coil domain of GOLGA5, oriented towards the cytoplasmic face of the Golgi (electronic supplementary material, table S5, figure 5*a*). Notably, the clustering of the three biotinylation sites within the sequence implies a close proximity of the N-terminus of GOLGA5 with NAA60.

Among the eight physical interactors of NAA60 suggested above, biotinylated peptide evidence was obtained for ABCD3 (K416) and AKAP1 (K66), in addition to GOLGA5, further supporting a more direct physical interaction of these three targets with NAA60 (electronic supplementary material, table S5). Of the 124 proteins identified with matching biotinylated peptide evidence in the NAA60 set-up but not in either of the two control set-ups, 35 proteins (out of the 57 proteins identified in common) were found to be enriched in the NAA60 versus eGFP control set-up (electronic supplementary material, tables S1 and S5). Interestingly, while not statistically significantly enriched in the NAA60 versus eGFP control set-up, biotinylation evidence (K3) of the N-terminal Nt-acetylated peptide (acetyl-T_2_KAGSKGGNLR_12_) of the NAA60 substrate leucine-rich repeat-containing protein 59 (LRRC59), which was also found to be regulated in expression upon si*NAA60* knockdown [19], was obtained in both NAA60 and SLC3A1 set-ups. This suggests that NAA60 substrates can nonetheless potentially be captured using BioID and also indicates that the substrate represents a shared SLC3A1/NAA60 proximal partner, indicating a significant and physiologically relevant overlap of the NAA60 and SLC35A1 proxeomes (electronic supplementary material, tables S15).

Intriguingly, of the N-terminal peptides previously found to be regulated in expression upon si*NAA60* knockdown, besides NAA60 biotinylated peptide evidence for LRRC59 and SEC61B (figure 5*b*), biotinylated peptide evidence was also found for Golgin subfamily B member 1 (GOLGB1, K3002, NAA60 set-up, alternative N-terminal peptide; Ac-M_40_EFNNTTQEDVQER_53_), Golgi resident protein GCP60 (ACBD3, K347, NAA60) (Ac-A_2_AVLNAER_9_), Vesicle-trafficking protein SEC22b (K169, NAA60 and SLC35A1 set-ups) (Nt-free V_2_LLTMIAR_9_), ancient ubiquitous protein 1 (AUP1, K359 and NAA60) (Ac-M_2_ELPSGPGPER_11_) and neutral amino acid transporter B(0) SLC1A5 (K502, NAA60 and SLC35A1) ((Ac-)M_1_VADPPR_7_) (while just below the cut-off, SLC1A5 was previously found to be reduced in its degree of Nt-acetylation (8%) following *NAA60* knockdown, and might thus represent a NAA60 substrate) (electronic supplementary material, table S5).

Since all six integral membrane proteins as well as the membrane-associated protein ACBD3 represent NAA60 proximal partners, and the observation that *NAA60* knockdown affects their N-terminal peptide abundance in either the cytoplasmic or organellar-enriched fraction [19], combined, these data hint at the possible mislocalization of these NAA60 proximal proteins and/or substrates upon *NAA60* knockdown.

### 2.3. Electron microscopy ultrastructural study of Golgi morphology in N-alpha-acetyltransferase 60 knockout cells

**Figure 6.**
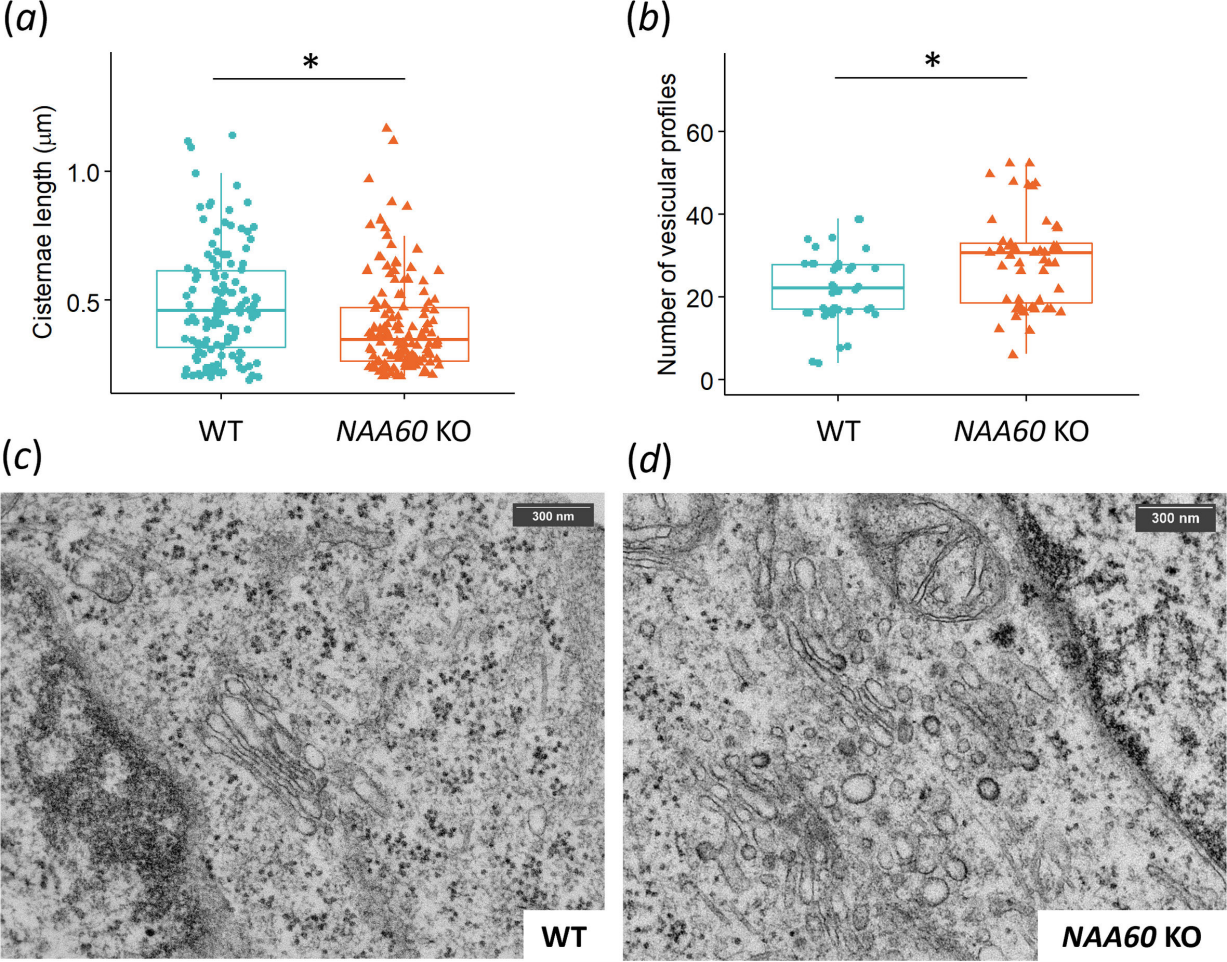
Ultrastructural analysis of Golgi fragmentation in *NAA60* KO HAP1 cells. (*a*) Boxplots showing the lengths of Golgi cisternae (µm) in *NAA60* KO and wild-type HAP1 cells. An asterisk indicates a significant difference in mean values, analysed using a linear mixed model with genotype (wild-type or *NAA60* KO) as a fixed effect and Golgi stack as a random effect (confidence level of 95%). (*b*) Boxplots depicting the number of vesicular profiles in *NAA60* KO and wild-type HAP1 cells. An asterisk denotes a significant difference in mean values determined by a two-sided *t*‐test (confidence level of 95%). (*c,d*) Representative TEM micrographs of wild-type HAP1 (*c*) and *NAA60* KO cells (*d*). Scale bars represent 300 nm.

Based on immunocytochemistry studies, it was suggested that ribbon unlinking (disrupted interactions between adjacent stacks) is a plausible explanation for the Golgi fragmentation into smaller and dispersed substructures observed upon NAA60 depletion [19]. However, a detailed study of Golgi structural changes upon NAA60 depletion is lacking. To gain detailed insight into the observed Golgi fragmentation, we assessed the effects of NAA60 deletion on Golgi ultrastructure using electron microscopy (EM) in CRISPR/Cas-generated HAP1 *NAA60* KO cells. Significant differences were observed in the lengths of Golgi cisternae, with shorter cisternae evident in *NAA60* KO cells (figure 6*a*). Additionally, an increased number of vesicle profiles [36] was observed in *NAA60* KO versus wild-type HAP1 cells (figure 6*b*). This suggests that cisternal maturation through vesicular shuttling between cisternae may be affected or increased, leading to fragmentation. Overall, these data partly support the hypothesis of Golgi-ribbon unlinking as originally proposed [19], but with additional effects on Golgi-cisternae stacking leading to vesiculation (figure 6*c*–d).

## 3. Discussion

Traditionally, mRNA translation has been thought to follow a spatial organization, with mRNAs encoding soluble proteins translated on free polysomes, while those encoding transmembrane or secreted proteins are being translated by ribosomes associated with the ER [37]. By juxtaposing NAA60 substrate data [19] with datasets on ER-localized translation [38], a pronounced preference for ER-localized mRNAs encoding known NAA60 substrates becomes evident (electronic supplementary material, figure S3). This compelling association between NAA60 and localized translation at the ER suggests a potential co-translational enzymatic function for NAA60.

Moreover, examination of N-terminal sequences of proteins synthesized at the ER reveals an enrichment of putative NAA60-type substrates [5]. Additionally, N-terminal proteomics data hints at increased expression levels of various ER protein translocon components following *NAA60* knockdown [19]. These components encompass key constituents of the protein-conducting Sec61 channel as well as complexes involved in ER nascent chain processing, such as the signal peptidase complex (SPase) and the oligosaccharyl-transferase complex (OST) [39]. Notably, as previously reported, TRAPγ, a recognized translocon accessory factor, exhibited the most significant difference in steady-state N-terminal acetylation levels upon si*NAA60*-mediated knockdown [19], further accentuating the potential connection between NAA60 and ER translocation complexes. These findings suggest a potential role for NAA60 in coordinating co-translational processes at the ER, including translocation and subsequent subcellular targeting of specific substrates.

In contrast to other NATs, NAA60 exhibits a predominant organellar localization, as recent investigations have revealed its peripheral membrane association on the cytosolic side of the Golgi complex, a localization facilitated by two amphipathic helices within the C-terminal region of NAA60 and membrane binding through a combination of hydrophobic and electrostatic interactions [18,19]. While evidence from NAA60 substrate analysis and ER-localized translation; therefore, suggests potential (transient) interactions with its substrates at the ER, subcellular localization studies, proxeome mapping and biotinylation topology data consistently support NAA60’s primary localization at the periphery of the Golgi.

Despite the scarce overlap between substrate data and proxeome data, the identification of LRRC59, a known NAA60 substrate, as a proximal partner of NAA60 hints at the feasibility of using BioID for NAA60 substrate capture. Such an effort aligns with the documented capacity of BioID to detect transient interactions and would be analogous to inactive catalytic domain capture technology, which facilitates the identification of enzyme substrates [40–42]. However, comparison of the proxeomes of wild-type and the catalytically inactive NAA60 point mutant, NAA60^Y97^ [43], did not reveal significant differences in proximal partners (data not shown), suggesting that loss of NAA60’s NAT activity did not substantially stabilize substrate interactions for their identification, nor did it impact the localization of NAA60 or its proximal partners, at least under conditions of overexpression (data not shown).

Collectively, in agreement with other Golgi-localized post-translational enzymatic activities [32,33], NAA60’s predominant organellar localization has led to the unfounded assumption of its post-translational action on substrate proteins. The scant overlap between previously identified NAA60 substrates and its proximal partners may; however, suggest that NAA60’s enzymatic activity is primarily restricted to the ER. These observations underscore the importance of exercising caution when interpreting localization or proxeome data in relation to enzymatic activities, particularly for enzymes like NAA60. Nevertheless, this does not exclude the possibility that NAA60 substrates may be translated at the ER and that NAA60 could facilitate post-translational protein targeting to membranes while concurrently acting post-translationally on its substrates.

Given the imprecise yet close co-localization of NAA60 with the *cis*-Golgi marker GM130 [20], we aimed to refine NAA60’s suborganellar localization and further differentiate neighbouring proteins from potential direct physical interactors by mapping the proxeomes of the Golgi marker proteins SLC35A1 and SLC35C2. Analysis of the expression and biotinylated protein patterns of the BirA*-fusion proteins revealed robust expression and specific biotinylation patterns. Microscopy-based examination of biotinylation staining displayed Golgi-like (GM130-like) patterns for NAA60 and SLC35A1. The observed staining patterns for SLC35C2 appeared more diffuse, suggesting an ER-*cis* Golgi-like localization pattern, consistent with literature reports on SLC35C2 localization. Subsequent BioID analysis revealed that the proxeome of NAA60 more closely resembled that of SLC35A1 compared with SLC35C2, indicating a more prominent localization of NAA60 in the *medial*/*trans*-Golgi compartment. In contrast, *cis*-Golgi and ER markers were found to be enriched in the proxeome of SLC35C2. Furthermore, a pairwise comparison of NAA60 with SLC35A1 seems to indicate that NAA60 spends less time at the ER compared with SLC35A1. Notably, NAA60 neighbours were enriched in proteins associated with other parts of the secretory pathway, such as endosomal proteins, overall suggesting a more predominant localization of NAA60 in the *medial*/*trans*-Golgi and later parts of the secretory pathway. Additionally, NAA60 was found in close proximity to numerous proteins related to the cytoskeleton, which are known to play a role in association with the secretory pathway. Interestingly, mitochondrial (MAVS, OCIAD1, AKAP1 and TDRKH) and peroxisomal proteins (MAVS and ABCD3) were also enriched in NAA60 proxeomes, consistent with the identification of some NAA60 substrates (e.g. phosphatidylglycerophosphatase and protein-tyrosine phosphatase 1 and sideroflexin-3) known to localize to these organelles [19]. Viewing the plasma membrane localization observed for *Arabidopsis* NAA60 [44], these findings suggest that NAA60 may have satellite localizations within and beyond the secretory pathway, potentially acting in trans through contact sites between organelles.

Interestingly, among the identified Golgi proteins in the proxeome of NAA60, numerous interactors serving critical roles in the assembly and membrane stacking of the Golgi cisternae and Golgi architecture and dynamics maintenance were identified, along with factors involved in intracellular transport regulation and the presentation of (a defined set of) transmembrane proteins.

Localized to distinct regions of the Golgi stack, central coiled-coil domain-containing golgins are typically anchored to the membrane through a C-terminal transmembrane domain or a domain binding a small GTPase, thereby making up part of the Golgi matrix (i.e. the proteinaceous material linking Golgi cisternae) and facilitating tethering interactions and Golgi-mediated protein trafficking [45]. Besides *cis*-Golgi GRASP65, NAA60-interacting *medial*/*trans*-cisternae localized GRASP55 is thus far the only of the two proteins that was shown to function in polarized Golgi stacking and represent other Golgi matrix constituents. Given the additional role of Rab proteins in regulating tethering processes and the cytoskeleton’s contribution to Golgi maintenance and functioning [46], golgins, Rab and GRASPS play a role in Golgi ribbon linking, supported by knockdown studies, as reviewed in [47–49]. This is particularly relevant considering the observed phenotype of Golgi ribbon unlinking and cisternae vesiculation in *NAA60* KO HAP1 cells, further contrasting the previously noted differential effects on Golgi integrity between *NAA60* knockdown and KO cells [20].

With the promising avenue of NAA60 BioID exploited to explore membrane interactions, differentiating between neighbours and physical interactors presents a challenge, and the use of suborganellar Golgi markers only partially addresses this issue. Therefore, additional binary interaction screens are warranted to validate NAA60 proximal proteins identified as true interactors of NAA60.

Consistent with recently reported primary familial brain calcification (PFBC) *NAA60* frameshift variants associated with NAA60 truncation and destabilization, the frameshifting 2 bp deletion (Asp40Leu) introduced in our *NAA60* KO HAP1 clones similarly leads to a significant truncation in the NAA60 protein, resulting in the deletion of the GNAT domain and rational loss-of-function, as demonstrated by *in vitro* NAT activity profiling of similarly truncated NAA60 variants [20]. While perturbed NAA60 N-terminal acetylation capacity may potentially be linked to PFBC, this does not preclude the possibility of perturbed localization of NAA60 proximal interactors, and thus not NAA60 activity *per se*, as a contributing or causative factor of the disease. Hence, caution is warranted when solely attributing *in vitro* NAA60 activity to *in vivo* substrates, as suggested for the SLC20A2 phosphate membrane transporter, whose gene has been previously linked to autosomal dominant PFBC [20]. Along similar lines, at least seven other solute carrier (SLC) transmembrane transporter proteins mediating solute influx and efflux across membranes (SLC35A2, SLC35E1, SLC1A5, SLC12A4, SLC3A2, SLC35B3 and SLC6A15) were identified as NAA60 proximal partners. Of these, at least four represent non-NAA60 substrates (i.e. two non-NAT substrates and two NatB-type substrates), indicating an association independent of Nt acetylation for many of the identified proximal partners. However, it is noteworthy that among these NAA60 proximal SLC proteins, the N-terminal peptide abundances of SLC35E1 and SLC1A5 were previously found to be regulated in either the cytoplasmic or organellar-enriched fraction of si*NAA60* knockdown cells, a finding consistent with the observed decrease in plasma membrane surface levels of SLC20A1 in *NAA60* KO cells [20], by and large suggesting a more general mislocalization of NAA60 proximal proteins upon *NAA60* knockdown or KO.

Consequently, further investigations are deemed necessary to assess whether NAA60 expression levels, which, when depleted, may result in disturbed localization of NAA60 interacting partners, and/or Nt acetylation activity *per se* are required for the restoration of Golgi integrity.

## 4. Conclusions

Our study and omics data integration shed further light on the multifaceted role of NAA60 in cellular processes. Jointly, available data suggest a co-translational enzymatic function and coordinating role for NAA60 at the ER, contrary to the prevailing assumption of its post-translational action on substrate proteins. Furthermore, our refined suborganellar localization analysis revealed a more prominent localization of NAA60 in the *medial*/*trans*-Golgi compartment. Interestingly, our findings also suggest potential satellite localizations for NAA60 within and beyond the secretory pathway, highlighting its potential diverse functional roles.

Moreover, the identification of proximal NAA60 proteins likely causatively linked to the Golgi fragmentation phenotypes observed upon NAA60 knockdown or KO provides further insights into the underlying mechanisms of NAA60’s actions. While impaired NAA60 Nt-acetylation capacity may be linked to disease pathology, our data further suggest potential perturbations in NAA60 complexation and altered localization of NAA60 proximal interactors, suggesting a broader role for NAA60 beyond Nt-acetylation.

In summary, our comprehensive analysis provides novel insights into the intricate functions of NAA60 in cellular physiology and disease. These findings pave the way for further research elucidating the molecular mechanisms of NAA60 and its potential therapeutic implications.

## 5. Methods

### 5.1. Cell culture

Human Flp-In™ T-REx™-293 cells (Thermo Fisher Scientific) were cultured in Dulbecco’s modified Eagle's medium (DMEM) containing GlutaMAX™ and 4.5 g l^−1^ glucose (Invitrogen, cat. no. 31966047). The HAP1 wild type and CRISPR/Cas9-edited human KO cell lines obtained from Horizon Genomics GmbH (Vienna, Austria) were grown in Iscove’s modified Dulbecco’s medium (IMDM) (Invitrogen, cat. no. 31980−048). Specifically, two h*NAA60* KO HAP1 clones (HZGHC003172c010 and HZGHC003172c003) were obtained, each containing an identical frameshift mutation caused by a 2 bp deletion in the fourth coding exon of NM_001083601 at genomic location chr16:34 79 477 (HAP1 NAA60 KO c117_118del:p.D40fs). This deletion was introduced using the guide RNA sequence TATCACGATACCATGAGTCT. The 2 bp deletion was confirmed by RNA-seq and Sanger sequencing, which was performed on PCR-amplified genomic DNA using the primers 5′-TTAGGTAGAGACTTAAGTGATGGGC-3′ (forward) and 5′-ACACACGTACGTACCTCTTTATGTA-3′ (reverse), with the latter also used as the sequencing primer (data not shown). Deletion of the aforementioned two nucleotides results in a frameshift and a premature stop codon, causing a significant truncation of the NAA60 protein, as demonstrated in [20].

All media contained 2 mM alanyl-l-glutamine dipeptide (GlutaMAX™) and were supplemented with 10% fetal bovine serum (FBS) (HyClone, Gibco™, cat. no. 10270106, E.U.-approved, South American origin), 50 units ml^−1^ penicillin and 50 µg ml^−1^ streptomycin (Gibco™, cat. no. 15070−063). Parent Flp-In™ T-REx™-293 cells were additionally supplemented with 15 µg ml^−1^ blasticidin (InvivoGen, cat. no. ant-bl-1) and 100 µg ml^−1^ zeocin for cultivation, with zeocin concentration increased to 800 µg ml^−1^ for counter-selection of stably transfected cells (InvivoGen, cat. no. ant-zn1). Stable transformants were maintained in media supplemented with blasticidin and 50 µg ml^−1^ hygromycin B Gold (InvivoGen, cat. no. ant-hg-1). Cells were cultured at 37°C in a humidified incubator with 8% CO_2_ and passaged every 3−4 days. Tetracycline-regulated expression of the transgenes in hygromycin-resistant stable cell pools was confirmed by doxycycline titration followed by anti-FLAG immunoblotting (data not shown).

### 5.2. Bacterial strains

For cloning purposes, *Escherichia coli* strain One Shot TOP10 chemically competent *E. coli* (Thermo Fisher Scientific) was used, following standard chemical transformation according to the manufacturer’s instructions.

### 5.3. Generation of Flag-tagged N-alpha-acetyltransferase 60 expressing construct

A construct expressing a Flag-tagged, wild-type, full-length NAA60 (Gene ID: 79903) was generated using standard restriction cloning and ligation techniques. The construct was created using the pMET7 vector with ApaI and SalI-HF restriction sites. PCR amplification of the full-length h*NAA60* coding sequence was performed from a previously reported ph*NAA60*-V5 construct in pcDNA3.1 [3] using primers designed to introduce ApaI and SalI restriction sites. The primer sequences were as follows: ApaI forward primer 5′-GCGAGGGCCCAGCTT**ATG**ACAGAGGTGGTGC−3′ and SalI reverse primer 5′-GCGAGTCGACCATGGTCCGGCTGTACTCG−3′. The correctness of the constructed vectors was confirmed by Sanger sequencing.

### 5.4. Immunocytochemistry and confocal microscopy

Stable human Flp-In™ T-REx™-293 cells were seeded in a chambered coverslip (µ-Slice 8 well, Ibidi) at a density of 20 000 cells per well in 200 µl of complete medium. Six hours after seeding, expression was induced with 1 µg ml^−1^ doxycycline (DOX) and 50 μM biotin. After 24 h, the cells underwent a two-step fixation process: initially, they were fixed for 30 min at room temperature with 4% paraformaldehyde (PFA) to achieve a final concentration of 2% PFA. Following the removal of the liquid, a second fixation was performed by adding 200 µl of 4% PFA to each well for 20 min at room temperature. The cells were then washed four times with PBS.

Permeabilization and blocking were carried out by adding 200 µl of permeabilization and blocking buffer (PBT buffer plus goat serum; i.e. 0.02% Triton X-100, 0.5% w/v BSA and 0.2% v/v goat serum in PBS) for 1 h. This was followed by incubation with the primary antibody diluted in PBT for 1 h at room temperature, and then the cells were washed four times in PBT. The cells were then incubated with a secondary antibody diluted in PBT for 1 h at room temperature and stained with DAPI (1 : 1000 dilution in PBS) for 30 min. Following three washes in PBS and a final wash in water, the cells were mounted using 1% *n*-propyl-gallate in glycerol and stored at 4°C in the dark.

The primary antibodies used included streptavidin-Alexa Fluor™ 680 conjugate (Invitrogen, S32358, 1/1000) and rabbit mAb to GM130 [EP892Y] (Abcam, ab52649). The secondary antibody was donkey anti-rabbit Alexa Fluor 488 (Life Technologies, 1/1000). Confocal images were acquired using a Fluoview FV1000 (Olympus) confocal microscope equipped with a 63×/1.4 NA HCX Plan-Apochromat oil immersion objective, approximately 1.2 airy unit pinhole aperture, and appropriate filter combinations, utilizing 405 diode and argon ion/argon krypton lasers.

### 5.5. Sodium dodecyl sulfate polyacrylamide gel electrophoresis and immunoblotting

Protein samples were prepared by adding sample loading buffer (Bio-Rad XT sample buffer) and reducing agent (Bio-Rad) according to the manufacturer’s instructions. Equal amounts of protein (40 µg), as quantified using the DC Protein Assay Kit (Bio-Rad), were separated on a 4% to 12% or 12% gradient XT precast Criterion gel using XT-MOPS buffer (Bio-Rad) at 150−200 V. Proteins were then transferred onto a PVDF membrane.

The membranes were blocked for 30 min using a 1 : 1 mixture of Tris-buffered saline (TBS) and Odyssey Blocking Solution (LI-COR, cat. no. 927−40003). Immunoblotting was performed by incubating the membranes overnight at 4°C with primary antibodies diluted in TBS-T (0.1% Tween-20) mixed with Odyssey blocking buffer. After three 10 min washes in TBS-T, membranes were incubated with secondary antibodies for 30 min in TBS-T mixed with Odyssey blocking buffer, followed by three washes in TBS-T and a final wash in TBS.

The primary antibodies and affinity reagents used were mouse anti-FLAG® (Sigma, F3165; 1/5000), mouse anti-GAPDH (Abcam, ab9484, 1/10000), and streptavidin-Alexa Fluor™ 680 Conjugate (Invitrogen, S32358, 1/2500). Secondary antibodies were IRDye 800 CW goat anti-mouse IgG (LI-COR, cat. no. 926−32 210, 1/10 000) and IRDye 800 CW goat anti-rabbit IgG (LI-COR, cat. no. 926−3221, 1/10 000). Protein bands were visualized using an Odyssey infrared imaging system (LI-COR).

### 5.6. Generation of stable, inducible expression BirA*-fusion cell lines of hNAA60, hSLC35A1 and hSLC35C2

h*NAA60*, h*SLC35A1* and h*SLC35C2*-expressing constructs for BioID were generated through Gateway cloning. Specifically, for h*NAA60*, the ph*NAA60*-V5 construct in pcDNA3.1 previously generated [3] served as a template to generate *attB*-flanked PCR products of *NAA60* using GoTaq polymerase (Promega). These products were suitable for a Gateway^®^ BP recombination reaction with a donor vector (pDONR221, Invitrogen, cat. no. 12536−017) to create entry clones. For amplification of *NAA60*, the forward primer 5′-GGGGACAAGTTTGTACAAAAAAGCAGGCTTCACCATGACAGAGGTGGTGCCATC-3′ (the start codon is underlined) and the reverse primer 5′-GGGGACCACTTTGTACAAGAAAGCTGGGTGCATGGTCCGGCYGTACTCG-3′ were used, with the reverse primer designed to fuse the PCR products in-frame with a C-terminal BirA*-FLAG tag encoded by the destination vector. h*SLC35A1* (orf 12918) and h*SLC35C2* (orf 12632) were obtained as entry clones in pDONR223 from the ORFeome resource [50].

The inserts from these h*NAA60*, h*SLC35A1* and h*SLC35C2* entry vectors were recombined into the pDEST-pcDNA5-BirA-FLAG-C-term [51] destination vector (provided by Dr Anne-Claude Gingras, Lunenfeld-Tanenbaum Research Institute, Toronto, Canada) using LR-clonase (Invitrogen, cat. no. 11791−020) following the manufacturer’s instructions. All constructs were sequence-verified by Sanger sequencing.

Stable cell lines expressing NAA60, hSLC35A1 or hSLC35C2 bait proteins in Flp-In™ T-REx™-293 cells, which contain a stably integrated flippase recognition target (FRT) site at a transcriptionally active genome locus, were generated as cell pools as described [52]. More specifically, Flp-In™ T-REx™-293 cells were co-transfected with a h*NAA60*, h*SLC35A1* or h*SLC35C2* expression construct and the Flp recombinase-encoding pOG44 plasmid (Invitrogen) in a 1 : 9 ratio (in six-well plates at 60−70% confluence) using Lipofectamine^®^ LTX with Plus™ Reagent (Invitrogen) in Opti-MEM according to the manufacturer’s instructions. Twenty-four hours post-transfection, cells were split into 10 cm plates to achieve a cell density of less than 25% confluence. Flp-FRT-mediated recombination of the inserts was selected in the presence of 15 μg ml^−1^ blasticidin and 50 μg ml^−1^ hygromycin B, with the selection medium replaced every 2−3 days until visible foci appeared. The selected cell populations were pooled by incubating with enzyme-free cell dissociation buffer (Thermo Fisher Scientific), collected by centrifugation (1000*g*, 5 min) at 4°C, and resuspended and maintained in complete DMEM supplemented with 15 μg ml^−1^ blasticidin and 50 μg ml^−1^ hygromycin B, along with penicillin/streptomycin. Cell pools were expanded and cultured without tetracycline. Stable cells expressing BirA*-FLAG fused to enhanced green fluorescent protein (eGFP) [53] were used as a negative control for the BioID experiments and were processed in parallel with the NAA60, hSLC35A1 and hSLC35C2 bait proteins (see §5).

### 5.7. BioID and enrichment of biotinylated peptides

For BioID experiments, stable cell lines were seeded at 1.3 × 10^7^ cells per 150 mm plate, corresponding to 70−80% confluence. Six hours after seeding, expression was induced with an optimized concentration of the stable tetracycline derivative doxycycline (DOX), which was set at 1 µg ml^−1^ for NAA60, SLC35A1 and SLC35C2 bait proteins and 0.75 ng ml^−1^ for eGFP bait expression. To limit overexpression effects and to obtain comparable bait expression levels, we optimized the DOX concentration used for each construct (data not shown). Sixteen hours post-induction, 50 μM biotin was added for an additional 24 h. Six 150 mm plates were used per construct for harvesting.

Cells were washed with D-PBS and collected by vigorous pipetting with 7 ml of ice-cold PBS to detach cells, followed by cell pelleting in two aliquots (5% and 95% of the cell material) at 600*g* for 5 min at 4°C. Pellets were stored at −80°C until further processing.

Frozen pellets corresponding to approximately 2 × 10^8^ cells (95% of cell material) were resuspended in 9 ml BioID lysis buffer (100 mM Tris-HCl pH 7.5, 150 mM NaCl, 2% SDS and 8 M urea) to reach a protein concentration of approximately 4 mg ml^−1^. This concentration was adjusted based on protein quantification using the DC Protein Assay Kit (Bio-Rad) on the 5% aliquot samples lysed in 50 mM Tris-HCl pH 8.0, 150 mM NaCl and 1% NP-40. The suspensions were subjected to mechanical disruption through three repetitive freeze–thaw cycles in liquid nitrogen and three sonication cycles of 2 min each on ice (25 s bursts at output level 4 with a 40% duty cycle using a Branson Ultrasonics™ Sonifier 250 Converter, Thermo Fisher Scientific). To the 9 ml lysate, 500 µl of a 50% suspension of pre-washed streptavidin agarose beads (Novagen, cat. no. 69203) was added (beads were washed three times by repeated resuspension and pelleting of the beads by centrifugation at 600*g* for 5 min with 2 ml of BioID lysis buffer), and the beads were incubated overnight with the lysate at room temperature on a rotator to capture biotinylated proteins. After overnight incubation, the beads were pelleted (600*g*, 2 min), and the unbound supernatant was removed and kept for further analysis. The beads underwent extensive washing, including four 5 min washes and a 30 min wash with 1 ml of BioID lysis buffer, a 30 min wash with high-salt buffer (100 mM Tris-HCl pH 7.5, 1 M NaCl), a 5 min wash with ultrapure water and three 5 min washes with 1 ml of 50 mM ammonium bicarbonate (pH 8.0).

Following the final wash, 90% of the beads were resuspended in 600 µl of 50 mM ammonium bicarbonate (pH 8.0), and 1 µg of mass spectrometry-grade trypsin (Promega, Madison, WI) was added.

The samples were incubated overnight at 37°C with vigorous mixing at 850 rpm. An additional 0.5 µg of trypsin was added the next day, followed by a further 4 h incubation. The beads were then pelleted (600*g*, 2 min), and the supernatant was transferred to a fresh low-protein-binding tube (Eppendorf). The beads were washed twice with 300 µl of HPLC-grade water, and these washes were combined with the original supernatant. The peptide solution was acidified with 10% formic acid to a final concentration of 0.2% and cleared of insoluble particles by centrifugation for 15 min at 16 000*g* (4°C). The cleared supernatant was transferred to clean tubes, vacuum-dried in a SpeedVac concentrator, re-dissolved in 25 µl of 2 mM tris (2-carboxyethyl)phosphine in 2% acetonitrile, and centrifuged for 15 min at 16 000*g* (4°C) before being transferred to mass spectrometry vials for LC-MS/MS analysis.

For the enrichment of biotinylated peptides, beads were washed three times with 300 µl of HPLC-grade water and the supernatant was completely removed using a micropipette. Biotinylated peptides were eluted by adding 300 µl of a solution containing 0.2% TFA, 0.1% formic acid and 80% acetonitrile in water, followed by vigorous mixing at 850 rpm for 5 min. The beads were then centrifuged at 600*g* for 5 min, and the first elution of biotinylated peptides was transferred to a clean Eppendorf tube. A second elution of 300 µl was boiled for 5 min to ensure maximum release of peptides from the beads and then combined with the first elution. The combined elution was dried in a SpeedVac concentrator, and the enriched biotinylated peptides were prepared for LC-MS/MS analysis as described above.

### 5.8. Liquid chromatography with tandem mass spectrometry analysis and data analysis of BioID samples

BioID samples were analysed by LC-MS/MS using an UltiMate 3000 RSLC nano HPLC system (Dionex) connected in-line to a Q Exactive instrument (Thermo Scientific, Bremen, Germany) as previously described [54,55]. The raw data files were processed using MaxQuant [56] with the Andromeda search engine [57] (version 1.6.10.43). MS/MS spectra were searched against the Swiss-Prot database (taxonomy: *Homo sapiens*), supplemented with sequences for BirA*Flag and eGFP. The search included potential contaminants from the contaminants.fasta file provided with MaxQuant. A precursor mass tolerance of 20 ppm was used for the initial search (for nonlinear mass recalibration) and 4.5 ppm for the main search. Trypsin was selected as the enzyme with an allowance for up to two (or three in the case of biotinylated peptide analysis) missed cleavages. Variable modifications included methionine oxidation and N-terminal protein acetylation. The FDR for peptide and protein identification was set to 1%, with a minimum peptide length of seven amino acids. The minimum score for both modified and unmodified peptides was set at 40. The ‘match between runs’ function was enabled, and proteins were quantified using the iBAQ algorithm [25]. In order to account for inter-replicate variability, replicate medians of iBAQ values were normalized before performing a two-group comparison.

For basic data handling, normalization, statistical analysis and annotation enrichment analysis, the open-source bioinformatics platform Perseus (version 1.6.15.0) [58] was used. Data visualization was performed using GraphPad Prism version 9.0.0. Perseus was used for non-supervised hierarchical clustering and 1D annotation enrichment using a two-sided test with the Benjamini–Hochberg FDR correction set at 0.01 or 0.02. Following the upload of the protein groups file from MaxQuant, data analysis proceeded as previously described [55]. Replicate samples were grouped, and iBAQ intensities were log(2) transformed. Proteins with fewer than three valid values in at least one group were removed, and missing values were imputed from a normal distribution centred around the detection limit (0.3 spread, 1.8 down-shift). A two-sample *t*‐test or multiple-sample ANOVA *t*‐test (FDR = 0.01, S0 = 0.1) was then conducted to detect enriched proteins across the different BioID setups.

### 5.9. Electron microscopy

Wild-type and h*NAA60* KO HAP1 cells were seeded at 6 × 10^6^ cells per 10 cm plate using 10 ml of medium. After 24 h, cells were fixed by removing 5 ml of medium and adding 5 ml of pre-warmed double-strength fixative solution (5% glutaraldehyde in 0.1 M sodium cacodylate, pH 7.4), followed by a 60 min incubation at room temperature. Subsequently, 5 ml of this solution was replaced with 5 ml of single-strength fixative (2.5% glutaraldehyde in 0.1 M sodium cacodylate, pH 7.4), and the cells were incubated overnight at 4°C. For the preparation of the samples for transmission electron microscopy (TEM), cells were washed three times with 5 ml of cacodylate buffer (0.1 M, pH 7.4) and scraped in the presence of 1.5% low melting point agarose (Sigma, cat. no. A4018) in cacodylate buffer followed by centrifugation (5 min at 800 g) in microcentrifuge tubes. After 30 min on ice, the pellets were cut off from the tube tips, cut into approximately 1 mm^3^ cubes with razor blades, and post-fixed in 1% osmium tetroxide in cacodylate buffer for 2 h in the dark, followed by a rinse in distilled water twice for 10 min each.

The pellets underwent dehydration in ethanol solutions of increasing concentrations (30%, 50%, and 70% for 10 min each at 4°C on a rotator). En bloc staining of samples was conducted with uranyl acetate in 70% ethanol for 30 min at 4°C. The process continued with dehydration in 90 and 100% ethanol concentrations. After treatment with propylene oxide (two changes of 15 min each at 4°C on a rotator), pellets were infiltrated and embedded in a 1 : 1 mixture of epoxy resin (Agar) and propylene oxide for 60 min, followed by overnight infiltration in a 2 : 1 agar : propylene oxide mixture. The next day, the pellets were embedded in Agar 100 and left to polymerize for 2 days after a 6−8 h incubation in a desiccator.

Ultrathin sections (50 nm thickness) were examined, and micrographs were taken using a JEOL JEM1400 transmission electron microscope at 80 kV.

The lengths of Golgi cisternae were evaluated using the method described by Sato *et al*. [36]. Golgi stacks (13–39) were blindly assessed in several sections from 8 to 30 cells across at least two independent experiments for each setup.

To measure the length of the cisternae, a line was drawn along the centre of each cisterna using the freehand tool in ImageJ (version 1.50) [59]. We classified the Golgi structures based on their dimensions: cisternae were identified as membrane profiles with a length at least four times their width. Cisternal remnants were defined as membrane profiles longer than 200 nm but not exceeding four times their width. Vesicular profiles were classified as membrane profiles with a length shorter than 200 nm.

The lengths of the cisternae were statistically analysed using a linear mixed model. This model accounted for the genotype (wild type or *NAA60* KO) as a fixed effect and the Golgi stack as a random effect. Analysis was conducted using Genstat for Windows 18^th^ Edition, with the significance level set at 5%. Additional statistical analyses and plotting were performed with GraphPad Prism version 6.01.

### 5.10. Software packages for statistics and data visualization

For basic data handling, normalization, statistics (if not stated otherwise), and annotation enrichment analysis, we used the freely available open-source bioinformatics platform Perseus (http://141.61.102.17/perseus_doku/doku.php?id = start) (v. 1.6.5.0). Perseus was used to visualize data from PCA, non-supervised hierarchical clustering and scatter plots. Furthermore, the 1D algorithm and Fisher’s exact tests implemented in Perseus [30] were used for annotation enrichment analysis. Heat maps, PCA plots and bar charts were generated using GraphPad Prism software (https://www.graphpad.com/). Protein sequences and transmembrane topology were automatically retrieved from UniProt and visualized using Protter [35].

## Data Availability

The mass spectrometry proteomics data have been organized in the Open Science Framework (OSF) project page [60] and are available at https://osf.io/f3g6j/ [61]. Supplementary material is available online [62].
